# A systematic review of the use of subcortical intraoperative electrical stimulation mapping for monitoring of executive deficits and neglect: what is the evidence so far?

**DOI:** 10.1007/s00701-021-05012-w

**Published:** 2021-10-21

**Authors:** Maud J. F. Landers, Margriet M. Sitskoorn, Geert-Jan M. Rutten, Emmanuel Mandonnet, Wouter De Baene

**Affiliations:** 1 Department of Neurosurgery, Elisabeth-TweeSteden Hospital, Hilvarenbeekse Weg 60, 5022GC Tilburg, the Netherlands; 2 Department of Cognitive Neuropsychology, Tilburg University, Tilburg, the Netherlands; 3 Université de Paris, Paris, France; 4 Institut du Cerveau (ICM), CNRS UMR 7225, INSERM U1127, Paris, France; 5 Service de Neurochirurgie, Hôpital Lariboisière, Paris, France

**Keywords:** Brain mapping, Direct electrical stimulation, Executive functions, Subcortical pathways, Tractography

## Abstract

**Background:**

Over the past decade, the functional importance of white matter pathways has been increasingly acknowledged in neurosurgical planning. A method to directly study anatomo-functional correlations is direct electrical stimulation (DES). DES has been widely accepted by neurosurgeons as a reliable tool to minimize the occurrence of permanent postoperative motor, vision, and language deficits. In recent years, DES has also been used for stimulation mapping of other cognitive functions, such as executive functions and visuospatial awareness.

**Methods:**

The aim of this review is to summarize the evidence so far from DES studies on subcortical pathways that are involved in visuospatial awareness and in the following three executive functions: (1) inhibitory control, (2) working memory, and (3) cognitive flexibility.

**Results:**

Eleven articles reported on intraoperative electrical stimulation of white matter pathways to map the cognitive functions and explicitly clarified which subcortical tract was stimulated. The results indicate that the right SLF-II is involved in visuospatial awareness, the left SLF-III and possibly the right SLF-I are involved in working memory, and the cingulum is involved in cognitive flexibility.

**Conclusions:**

We were unable to draw any more specific conclusions, nor unequivocally establish the critical involvement of pathways in executive functions or visuospatial awareness due to the heterogeneity of the study types and methods, and the limited number of studies that assessed these relationships. Possible approaches for future research to obtain converging and more definite evidence for the involvement of pathways in specific cognitive functions are discussed.

**Supplementary Information:**

The online version contains supplementary material available at 10.1007/s00701-021-05012-w.

## Introduction

Over the past decade, the functional importance of white matter pathways has been increasingly acknowledged in neurosurgical planning. Novel subcortical pathways have been identified by new neuroimaging and tractography techniques [38, 43]. Additional information on the functionality and clinical relevance of these pathways has come from lesion-symptom and brain mapping studies [8, 24, 84].Together, these anatomical and functional findings contributed to a shift from a predominantly localized view on functional topography towards a more network-based view. Specific brain functions are no longer solely attributed to a certain location on the cortex, but are thought to be represented by a particular network of cortical areas that are connected via subcortical pathways [8, 15, 20, 38, 41, 43, 73].

A method to directly study anatomo-functional correlations is direct electrical stimulation (DES). DES has been widely accepted by neurosurgeons as a reliable tool for the identification of functional areas during surgery. It is currently considered the clinical gold standard for this purpose [50]. Historically, DES has been predominantly used to identify motor and language cortical areas [25, 30, 32, 49, 72]. In the past decades, it has also been routinely used to map the subcortical anatomy [9], and, more recently, to map cognitive functions other than language, such as executive functions and visuospatial awareness [23]. The rationale for intraoperative mapping of these latter functions is that cognitive and visuospatial impairments occur relative frequently among patients with glial tumours, both before and after surgery [69]. Several studies demonstrated that executive function impairments three months after surgery in glioma patients are frequent [14, 61]. More specifically, a recent study showed that long-lasting executive function impairments in low-grade glioma patients are related to surgical disconnections of frontal and parietal white matter pathways [17]. Patients with executive functions impairment exhibit for example difficulties with planning, initiation, regulation and verification of complex, goal-directed behaviour [40] which negatively impact normal daily, social and professional life [21, 26, 55] and health-related quality of life [1]. Patients with visuospatial deficits also exhibit a diminished postoperative quality of life [22, 39, 60].

The nomenclature of executive functions is widely discussed in the literature, but these functions can generally be described as ‘a set of general-purpose control mechanisms that regulate the dynamics of human cognition and action’ [53]. There is a general agreement that the following three mutually correlated, but distinct core executive functions can be distinguished: (1) inhibitory control, (2) working memory, and (3) cognitive flexibility [21, 28, 53, 54]. (1) The first core executive function, inhibitory control, is the ability to control oneself and the ability to control attention. Both are endogenous, top-down processes necessary for active, goal-driven execution of cognitive functions [34, 65, 85]. Self-control involves the inhibition of impulses, old habits, thoughts of action and external stimuli, and allows one to change and choose our reactions. Deficits in inhibitory control of oneself may for example become manifest as the so-called frontal syndrome (i.e., deprecated term used for psychopathological conditions after frontal lesions) [2, 83]. Inhibitory control of attention is required for selective attention and focus, and to suppress or ignore interfering stimuli based on one’s intentions or goals. Deficits in inhibitory control of attention may impair the distribution of directed attention, and may lead to concentration difficulties and sensory overload [85]. (2) The second core executive function, working memory, is generally described as a temporary storage system in which information can be processed and manipulated in a goal-driven context [3]. There are two types of working memory: verbal working memory, necessary for spoken and written language, and visuospatial working memory, necessary for recognition and processing of visual and spatial information [80]. Deficits in working memory may result in anterograde amnesia and lead to problems with, for example reasoning, decision-making and behaviour in general [5, 21]. Working memory and inhibitory control generally co-occur, as working memory is needed to remember what is relevant and what should be inhibited (e.g., concentrating on a goal). Vice versa, inhibitory control is necessary for selection and priority setting (e.g., ignoring distractions), which are needed to direct information for temporal storage in working memory [21, 80]. (3) The third core executive function, cognitive flexibility, is the ability to change perspective (i.e., to shift attention) by adapting to a new set of norms, demands or priorities. This function is needed to switch between tasks. Deficits in cognitive flexibility are related to a variety of attentional problems, for example difficulties to concentrate and oversensitivity [88, 90]. These deficits are often reported in brain tumour patients, and more generally, in patients with brain damage [29, 31]. Cognitive flexibility requires inhibition to deactivate the prior perspective and activation of the new perspective in working memory, demonstrating the commonality of the three executive functions [21].

Visuospatial awareness refers to the ability of identifying, processing and interpreting visual information about objects in space. Multimodel cognitive deficits due to lesions in several different networks (e.g., motor control network, ventral attention network) may lead to a reduction or loss of spatial awareness for the contralateral space, clinically resulting in ‘neglect’[7, 11]. It has been hypothesized that in hemispatial neglect a one-sided attentional bias occurs due to inhibition of the contralateral hemisphere. Note that the underlying spatial, inhibitory and attentional networks of neglect have not yet been fully elucidated [6, 36, 57, 87]. However, neglect often co-occurs with executive functional deficits and it is hypothesized that neglect may occur as a consequence of lesions to several regions involved in different executive networks [39]. Further evidence for this is found in rehabilitation studies, which demonstrate that rehabilitation programs that focus on spatial attention have not been able to alter most of the perceptual biases present in neglect, whereas rehabilitation strategies that focus also on spatial working memory capacity and executive control have been able to alter the perceptual biases present in neglect [81].

Several groups have started to use tasks to monitor executive deficits and neglect during surgery using DES [27, 67, 78, 95]. However, there is no consensus yet on intraoperative task protocols that are best suited for this purpose. This review summarizes the evidence so far from DES studies on subcortical pathways that are involved in executive functions and visuospatial awareness. This knowledge can guide neurosurgeons in their current practice, and is a first step towards a consensus protocol for cognitive monitoring.

## Methods

### Literature search

A systematic literature search was conducted to identify studies in which subcortical mapping during awake neurosurgical operations was used to investigate tracts involved in inhibition, working memory and cognitive flexibility. The databases Pubmed Central, Embase, Web of Science, and Cochrane Central were searched up to July 19, 2020. Search terms were established by the first author and were verified by the second and last author. Search strategies were built and performed by the first author (see Supplemental material 1). Inclusion criteria were studies investigating adult patients, with any type of brain tumour, who underwent awake surgery, in which direct electrical stimulation of subcortical pathways was performed, and by whom neuropsychological performance was measured. Eligible studies were clinical trials, randomized controlled trials, case series and case reports from all years of publication, written in English. Systematic reviews, narrative reviews and meta-analyses were also included initially. Letters, (conference) abstracts, and protocols were assessed as ineligible. Based on the results of our initial search we have added neglect to the search strategy.

### Study selection

Studies were screened on title and abstract by the first and last author. Full text was screened if it was not clear from the abstract whether the study met the inclusion criteria. Screening results from both authors were compared and cases of doubt were discussed. Consensus was reached for all cases. Reviews and meta-analyses and reference lists from eligible articles were screened by the first author for additional articles.

### Assessment of executive functions and visuospatial awareness

To ensure that all studies investigating executive functioning would be covered in the search strategy, a broader term and its derivatives were also added: neuropsychological performance, and cognition. In this way, we prevented studies assessing more than one cognitive function from being mistakenly excluded. Therefore, all abstracts were critically evaluated by the first and last author to examine whether inhibition, working memory, cognitive flexibility (or related terms), or visuospatial awareness were mentioned, even if this was not the main topic of the study. Note that studies examining higher cognitive functions that do not require executive control or visuospatial awareness were therefore not included in this review.

### Quality assessment of studies

The quality of each of the included studies was assessed by the first and last author using the 8-item Methodological Index for Nonrandomized Studies (MINORS) for non-randomized studies [79]. In case of a comparative study, 4 additional items were scored. MINORS includes the assessment of risk of bias in individual studies. Consensus was reached for all cases.

## Results

### Study selection

**Fig. 1:**
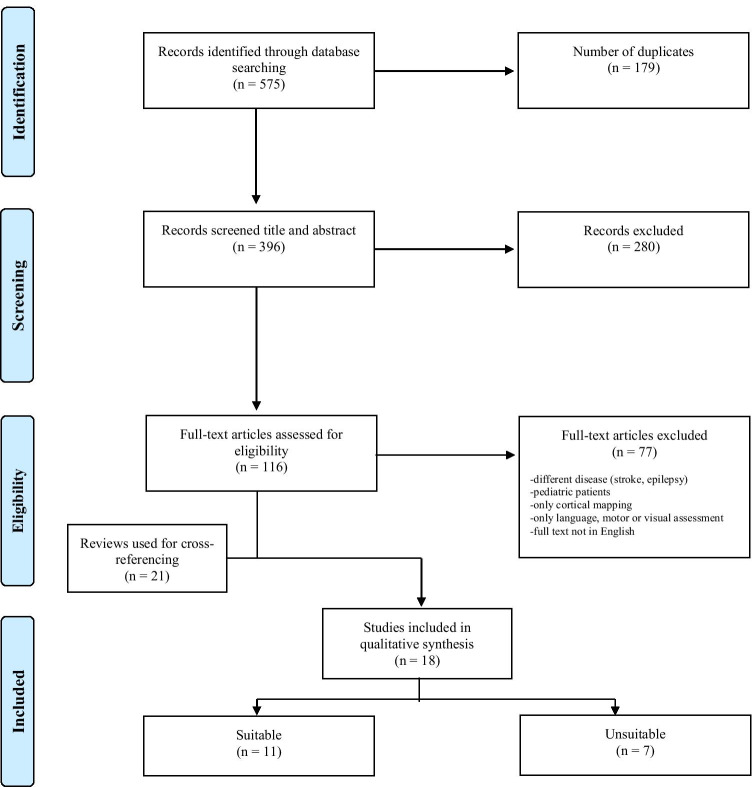
PRISMA flow diagram [51] presenting the selection of studies for DES of subcortical pathways involved in executive functions

The initial systematic literature search identified 575 articles (see Fig. 1) [56]. After removing duplicates and screening titles and abstracts, 116 articles remained that met the inclusion criteria. Full-text articles were assessed for eligibility, resulting in the inclusion of 18 articles. Twenty-one reviews were used for cross-referencing but this did not lead to the inclusion of additional articles, nor did screening the reference lists of the eligible articles.

### Study characteristics

Of the 18 eligible studies 11 were deemed to be suitable for this review because they provide a reliable estimate of the stimulated anatomical pathway, as well as the behavioural deficits during electrical stimulation mapping. The suitable included studies were conducted between 2005 and 2019, of which 4 were prospective mapping studies, 3 were case reports or case series, 3 were retrospective studies and 1 compared two cases with a control group. The results are presented in Table 1 and summarized below per function. The column ‘Tract’ includes all the tracts tested (positive and negative) for the functions. Two studies were deemed unsuitable because they assessed recognition [12, 51]. The other five studies that were deemed unsuitable did assess the functionality of subcortical pathways in executive functions or visuospatial awareness, but did not clearly indicate which subcortical pathway was stimulated and were therefore not considered suitable to answer the research questions of this review. All five unsuitable studies reported on inhibitory control and used the line bisection task [18, 59, 74, 75] or target cancellation task [18] to assess visuospatial awareness or an intraoperative Stroop test to assess inhibition of automatic responses^44^.

**Table 1:** Overview of studies that used DES to assess the functionality of subcortical pathways in executive functions. Only those studies are included where a reliable estimate could be made of the stimulated anatomical pathway as well as the behavioural deficits during electrical stimulation mapping

Study	Type	Neuropsychological (dys)function	Method	Task	Incorrect response	Tract	Method(s) used to identify stimulated tract(s)	Intraoperative results	Authors’ conclusion
**Burks et al. (2017) **[12]	Retrospective comparison study (standard surgery *n* = 25 vs. awake surgery with subcortical mapping to preserve cingulum *n* = 15)	Attention: Switching from undirected thinking to attention-driven, goal-directed thinking	15 patients with anterior butterfly gliomas underwent awake subcortical mapping	Task requiring praxis, bimanual coordination and attention, e.g., playing musical instrument, assembling auto parts, knitting If possible while simultaneous naming objects to increase sensitivity	Inability to perform task Inability to execute naming while performing the task	CINGULUM in anteromedial frontal lobe	Neurosurgeon identified cingulum intraoperatively Preoperative DTI tractography was used to reconstruct cingulum postoperatively and was compared to post mortem fibre tract dissections	*No details provided*	Anterior butterfly gliomas can be safely removed using a novel attention-task-based awake brain surgery technique that focuses on preserving the anatomical connectivity of the cingulum and relevant aspects of the cingulate gyrus
**Herbet, Yordanova, Duffau (2017) **[30]	Three cases report	Spatial attention: neglect	3 patients with right temporal glioma	Line bisection task	Rightward deviation from midpoint	IFOF right	In absence of preoperative diffusion data, a white matter atlas was used to calculate the disconnection probability for each tract using Tractotron software (BCBToolkit)	Stimulation of IFOF around the roof of the inferior horn of the lateral ventricle (patient 1 and 2) and along the middle-to-posterior part of the IFOF (patient 3) resulted in significant deviations from the mean	In conclusion, we provide preliminary evidence for the causal role of the right IFOF in spatial attention. In general way, our findings add support for both the multilayer and multifunction view of the IFOF, which has been recently demonstrated to subserve other cerebral functions
**Motomura et al. (2018) **[58]	Retrospective analysis to define functional boundaries (*n* = 9) including pre- and postoperative neuropsychological assessment	Verbal working memory Spatial working memory Spatial awareness	9 patients with diffuse frontal low grade gliomas	*Digit span test (forward and backward)* *N-back test (1-back and 2-back)* *Double task* Standard line bisection task	*No details* *No details* Lateral midline deviation	*FAT left and right* *IFOF left* FST right	Postoperative scans used to identify tracts Tractography technique not defined	*In 6 out of 9 patients positive sites were found* *Unclear which task was used for which subcortical tract* No positive responses in any patient	We were able to preserve higher neurocognitive functions (apart from language/motor), including working memory and spatial cognition during awake brain mapping in patients with nondominant right frontal tumours
**Papagno et al. (2017) **[56]	Prospective awake mapping study (*n* = 29), incl pre- and postop assessment, subcortical mapping in (*n* = 9)	Verbal short term memory	9 patients with a tumour in left dominant hemisphere	Digit span forward	*Item errors*: Substitutions of an item with a different one, omissions and intrusions *Order errors*: correct item in wrong position	SLF-III left	Preoperative DTI scans used for deterministic tractography (Trackvis)	Significantly less correct sequences and more item and order errors during stimulation of SLF-III compared to stimulation of long segment of the AF	Subcortical stimulation of the SLF-III (anterior segment of the indirect AF pathway) interferes with digit span, producing significantly more order than item errors
**Kinoshita et al. (2016) **[32]	Voxel-based lesion-symptom mapping (*n* = 24) and awake mapping study (*n* = 2)	Spatial working memory Spatial awareness	2 patients with prefrontal glioma	Spatial 2-back task Standard line bisection task	Incorrect response in 2 or 3 sets Rightward deviation	SLF-I right	Pre- and postoperative DW-MRI scans for DTI tractography	DES reproducibly caused difficulty in providing correct responses in the 2-back task No deviations during stimulation	Right prefrontal glioma resection can result in chronic spatial WM deficit, potentially caused by an impairment of the dorsal frontoparietal subcortical white matter pathway subserved by SLF I
**Roux et al. (2011) **[64]	Prospective mapping study (*n* = 50), with subcortical mapping (*n* = 18) and preoperative fibre tracking (*n* = 8)	Spatial neglect	18 patients with various brain lesions	Standard line bisection task	Line deviation	SLF-II right IFOF right AF right ILF right	Preoperative DTI in 8 patients, postoperative DTI in 6 patients used for deterministic tensor line method using Sisyphe software (self-developed)	SLF-II: 1/22 stimulations leftward deviation IFOF: 1/18 stimulations leftward deviation AF: 4 rightward, 1 leftward deviation/35 stimulations ILF: 0/26 stimulations	Sparing the superior longitudinal fascicle II and the inferior occipitofrontal fascicle was essential to avoid neglect in operated patients. No induced line deviation was detected when stimulating the region of the inferior longitudinal fascicle
**Thiebaut de Schotten et al. (2005) **[78]	Two case reports	Spatial awareness: left unilateral neglect	2 patients with low grade glioma (right temporal lobe and right inferior parietal lobule)	Standard line bisection task	Rightward deviation	SLF II right	Postoperative DTI scans. Tractography technique not defined	In 1 case rightward deviation was found upon subcortical stimulation	Parietal-frontal communication is necessary for the symmetrical processing of the visual scene
**Vallar et al. (2014) **[80]	Prospective mapping study with pre- and postoperative assessment and tractography (*n* = 7)	Left visuospatial neglect	7 patients with glioma (six right hemisphere, one left hemisphere)	Computerized line bisection task	Rightward deviation	Right SLF-II SLF-I SLF-III IFOF	Preoperative and postoperative DTI scans used for preoperative fibre assignment by continuous tracking (FACT method) and verified by postoperative deterministic and probabilistic tractography (FMRIB’s Diffusion Toolbox)	In all 6 patients with right hemisphere gliomas rightward deviation with stimulation of right SLF-II No deviation with stimulation of SLF-I, SLF-III, IFOF	The SLF II is involved in the orientation of spatial attention
**Puglisi et al. (2019) **[59]	Prospective, mapping study with Stroop test (*n* = 34) and control (*n* = 29) combined with lesion-symptom mapping of resection cavities and postoperative performance (*n* = 63)	Interference control/executive functioning	34 patients with frontal right hemisphere gliomas	Stroop Test	Performance errors (naming color instead of word or with a delay > 1 s) on three non-consecutive stimulation trials	Inferior FST right Anterior thalamic radiation right	Postoperative whole brain tractograms (HARDI) of 8 patients used for spherical deconvolution modelling and deterministic tractography (StarTrack software)	In 25 (of 34) patients, interference during stimulation of the periventricular white matter medial to the right IFG and lateral and superior to the striatum (34 positive sites)	The intraoperative data combined with tractography suggests that corticosubcortical tracts, over corticocortical connections may be vital in maintaining efficiency of cognitive control processes
**Mandonnet et al. (2020) **[43]	Case report	Set shifting ability	1 patient with a low-grade glioma in the right supramarginal gyrus	Trail making test part B	Disturbances in performance in at least two consecutive trials under active stimulation	Parts of the arcuate fasciculus, MLF, ILF, IFOF and corpus callosum	Postoperative diffusion MRI scans used for constrained spherical deconvolution followed by probabilistic tractography using a home-made pipeline	Intraoperative impairment on TMT-B performance during the transient disruption of white matter fibres passing through the right parieto-temporal junction	The data support the need of a network-level approach to identify the neural basis of the TMT-B and point to the Control network B as playing an important role in set-shifting
**Rolland et al. (2018) **[63]	Retrospective mapping study (*n* = 14), neglect assessed in 5 patients	Spatial awareness	5 patients with a glioma in the right inferior parietal lobe	Standard line bisection task	Rightward deviation	Right SLF-II Right AF	No diffusion scans obtained from patients. Tractography-based white matter atlas was used to get indication of relationship between white matter tract and stimulation sites	Stimulation of SLF-II in 4 patients and of AF in 1 patient led to rightward deviations	This surgical series focuses on right IPL gliomas. The complex functional connectivity detected within and around this region fully supports the use of intraoperative multimodal functional mapping for optimizing outcomes

#### Inhibitory control

Only one of the eleven included studies reported on subcortical pathways involved in inhibitory control. This study investigated inhibitory control of oneself through performance on the Stroop task during subcortical electrical stimulation in 34 patients [66]. Their stimulation results indicate that the right inferior frontostriatal tract (FST) and the right thalamic radiation are involved in inhibitory control.

#### Working memory

Two of the eleven included studies reported on pathways involved in working memory. One study reported on visuospatial working memory and showed that subcortical stimulation of the right SLF-I led to incorrect responses on a spatial N-back test in two patients. Note that this study also examined inhibitory control. The other study reported on verbal working memory and showed that subcortical stimulation of the left SLF-III in nine patients resulted in interferences with the digit span [63].

#### Cognitive flexibility

Two of the eleven included studies reported on pathway involvement in cognitive flexibility. One study concluded that stimulation of the cingulum leads to problems in the execution of a double task in 15 patients, although no intraoperative stimulation results were reported [13]. The other study was a case report in which set-shifting ability was investigated with the trail making test part B (TMT-B), showing that stimulation of white matter fibres passing through the right parieto-temporal junction leads to impaired performance on the TMT-B [48].

#### Visuospatial awareness

Seven of the included eleven studies investigated visuospatial awareness with a line bisection task and regarded deviation from the midpoint as an incorrect response indicating neglect. Incorrect responses were mainly found when stimulating the superior longitudinal fasciculus (SLF)-II. Across all studies that assessed right SLF-II, stimulation resulted in neglect in all 11 patients [70, 86, 89], suggesting that this pathway is involved in spatial attention. Negative responses (i.e., no intraoperative error on the task) were obtained from stimulations of the right SLF-I in 8 patients [35, 89], the right SLF-III in six patients [89] and the right FST (number of patients unknown) [58] suggesting that these tracts are not involved in spatial attention. Stimulation results for the right inferior fronto-occipital fasciculus (IFOF) and the right arcuate fasciculus (AF) are inconsistent and do not allow to make general conclusions. One study reported that electrical stimulation of the right IFOF resulted in neglect in 3 patients [33], whereas another study reported no neglect after stimulation of the right IFOF in six patients [89]. In another study the AF was only stimulated in one patient and resulted in neglect [70]. One study performed subcortical mapping of the right SLF-II, the right IFOF, the right AF and the right ILF in 18 cases [71], but only reported the number of positive and negative stimulations per pathway and not in how many patients they occurred, which complicates the interpretation of the results.

### Quality assessment

The mean 8-item MINORS score for the eleven included studies varied between 6 and 12, mean 8.82 and standard deviation 1.75 (see Supplemental material 2). Three of the eleven studies were case-reports or case-series and could not be scored adequately on item 2, 7 and 8. Only four studies had an adequate control group, and the mean 12-item MINORS score for those comparative studies varied between 11 and 18, mean 15.60, and standard deviation 2.50. None of the studies reported on unbiased assessment of the study endpoint or on a prospective calculation of the study size.

## Discussion

This systematic review aimed to determine the evidence so far from electrical stimulation studies on the involvement of subcortical pathways in executive functions and visuospatial awareness in patients with brain tumours. The results uncovered that the evidence from the few eligible studies was not sufficient to unequivocally establish the involvement of any subcortical pathway in higher cognitive functions. We found only eleven studies that explicitly clarified which subcortical tract was stimulated during electrical stimulation, and were, therefore, suitable for further analyses. Study characteristics, methods and tasks were heterogenous, and methodological quality varied. Clearly, neurosurgical groups are exploring new methods to assess cognitive functions during brain tumour surgery, but are far from consensus on protocols and indications. This complicated the comparison and summarization of findings. However, after reviewing the literature, the results indicate that the right SLF-II is involved in visuospatial awareness, the left SLF-III and possibly the right SLF-I are involved in working memory, and the cingulum is involved in cognitive flexibility. These (preliminary) findings do not only reflect the urge for more research on subcortical mapping of executive functions, but do also provide an important first step towards establishing the potential functional important pathways for neurosurgical practice.

### Inhibitory control

We defined inhibitory control as the ability to control oneself and the ability to control one’s attention, needed to suppress goal-irrelevant stimuli and responses. A commonly used test to assess resistance to interference is the Stroop Colour and Word test [82]. Although several studies used this test to identify areas involved in interference control during cortical mapping [67, 95], only one study investigated performance on the Stroop task during subcortical electrical stimulation and indicate that the right inferior FST and the right thalamic radiation are involved in interference control [66].

### Working memory

We defined working memory as a temporary storage system, needed to process and manipulate verbal and visuospatial information in a goal-driven context. Our results suggest that the left SLF-III is involved in verbal working memory and that the right SLF-I is possibly involved in visuospatial working memory. However, the one study that claimed involvement for the left SLF-III in verbal working memory only used a forward digit span task [63], suggesting impairment of recall rather than of executive functions [4, 96]. Evidence from fMRI-studies in traumatic brain injury patients and brain tumour patients suggests that working memory deficits are the result of dysfunction of networks [76, 77]. When performing a working memory task, one has to switch from a resting state (i.e., default mode network) to a task-related active state (i.e., central executive network). As the SLF connects frontal and parietal areas of and within both these networks in both hemispheres [35, 37, 63], the SLF may play a central role in both verbal and spatial working memory. The fact that the SLF has three branches that connect different areas and that the SLF has different functional specializations for each hemisphere might explain why different functions were found for this tract [10, 16, 44].

### Cognitive flexibility

In this review, cognitive flexibility is defined as the ability to change perspective by adapting to a new set of norms, demands or priorities, which is needed to switch between tasks. Only two studies have reported on cognitive flexibility and their results suggest involvement of the cingulum and white matter fibres that cross the right parieto-temporal junction. A possible reason why cognitive flexibility is not often studied during awake neurosurgical procedures is that tests that measure this function are not particularly suited for the intraoperative setting. Although different forms of task switching tests have been used to measure cognitive flexibility [34, 92], these tests all rely on an ongoing sequence of task repeats and task switches, in which prolonged reaction time or making the wrong switch indicate problems in cognitive flexibility. Given that stimulation typically only lasts for four to six seconds, the ongoing aspect of task cues and the fact that stimulation may interfere with any presented cue, makes it very difficult to use this type of paradigm to measure cognitive flexibility during awake neurosurgical procedures.

Some studies use the TMT-B as a measure of cognitive flexibility and suggest this test is feasible for intraoperative use [45, 64, 68]. However, the TMT-B might not always be sensitive enough as a measure of cognitive flexibility. Complete disruption of cognitive flexibility will result in a shifting error that can be measured with the TMT-B. However, reduced cognitive flexibility will result in an increased reaction time on shifting trials, that can only be adequately interpreted if assessed together with the TMT part A (TMT-A). A recent review concluded that alternating task performance primarily correlates with the TMT-B to TMT-A ratio (time measures). However, measuring both these tasks during surgery and using the ratio as a measure for performance at any given site, seems very challenging from a practical point of view [93]. Furthermore, another recent study found that TMT-B performance is greatly influenced by processing speed, just like the performance on shifting attention tasks, which complicates intraoperative use, where processing speed is already affected by the setting [42]. Additionally, the timing of stimulation and the stimulation location may distort performance on the cognitive flexibility task only through its effect on processing speed.

### Visuospatial awareness

Deficits in visuospatial awareness may result in a disorder called neglect, which is defined as a disturbance in the spatial distribution of directed attention [52]. This disorder is often seen as a consequence of right parietal brain lesions that cause neglect in the left hemispace [19]. Current knowledge from electrical mapping studies suggests that the right SLF-II and possibly the right IFOF are involved in visuospatial awareness, whereas the right SLF-I, the right SLF-III and the right FST are not. Remarkably, in all but two studies [70, 89], the authors who described positive findings drew rather firm conclusions, as they stated that the subcortical tracts in question are ‘necessary for’ or ‘essential for avoiding’ neglect, while results were not clearly described or were only found in a single patient.

### Identification of stimulated tracts

An important point when claiming functionality of a given tract is to know exactly what tract was electrically stimulated. The studies included used different methods to determine the subcortical white matter tracts that were stimulated (see Table 1). The methods varied from identification based on the neurosurgeon’s anatomical knowledge, to identification with the help of navigated tractography. A couple of studies combined preoperative and postoperatively acquired tractography to reconstruct intraoperative stimulation sites and their spatial relation with fibre pathways [71]. Others used additional lesion-symptom mapping or disconnection analyses (to identify overlap with a white matter tract) [13, 35, 48, 67]. However, none of these methods is free of (significant) error. Tractographic results are variable due to a great variety in techniques, anatomical uncertainty (physiological differences, disease related differences, undiscovered pathways), and a lack of standardization per method, because results depend on parameters chosen by the user [91]. Furthermore, inaccuracies of neuronavigational systems and additional intra-operative brain-shift may lead to errors in the documented spatial locations of the mapping sites [62]. Consequently, the possibility of inaccurate findings, false negatives and positives, and irreproducibility of stimulation results complicates the determination of anatomo-functional correlations. It addresses the need for consensus on an intraoperative mapping protocol for both stimulation and documentation of responses.

### Limitations

We chose to present and discuss the results per cognitive function. However, all cognitive functions have a certain overlap in function and neurobiological underpinning, which complicates the interpretation of subcortical stimulation results. This review uses data that are acquired during neurosurgical procedures, in a setting that is obviously very different from a regular neuropsychological testing environment and only allows for one single-moment assessment (and not for assessment of performance over time). The tasks used for the assessment of neuropsychological functions are not developed or validated for intraoperative use and normally lack ecological validity (i.e., translatability to daily life functioning). Moreover, there are few sites in the brain where one can selectively target a single pathway and these pathways are clearly part of (one of more) networks that as a whole perform specific functions. Hence, stimulation of a single tract (if possible) may result in disruption of distant networks, whilst the neuropsychological findings are attributed to the tested tract for understandable but incorrect reasons. Furthermore, for most long range associative tracts there is still a substantial variability in the nomenclature and taxonomy (e.g., AF and SLF [94]), which may again lead to an incorrect attribution of function to tract [47]. In addition, the stimulation responses also depend on the tissue that has already been resected. Although this is not necessarily a problem from a surgical point of view, it makes it difficult to assess the exact functionality of a tract. Furthermore, we noticed that most studies reported only the positive mapping sites (i.e., sites where errors were found during mapping) and not the so-called negative mapping sites. We believe that strict documentation of negative mappings is also necessary to validate hypotheses for specific white matter tracts. Finally, the methods and quality of the studies varied, and the different study types could not always be compared. Consequently, due to this heterogeneity that limited the comparison between studies, every study had to be placed in the correct perspective by the authors, and induced a potential risk of bias across studies.

### Future research

When summarizing the findings from subcortical mapping studies and carefully considering their limitations, as well as the limitations of this study, it is evident that DES can provide us with unique and important information regarding the functionality of the subcortical pathways through the localization of transient functional deficits. It has the potential to move forward both clinical practice and neuroscience. However, the combined results from published DES studies are at the moment not sufficient to unequivocally determine the involvement of subcortical pathways in executive functions or visuospatial awareness. Only when cognitive tasks are carefully introduced into the operating room, DES will be able to function as a reliable tool to increase the safety of brain tumour resections. Recently, a roadmap was proposed that addresses the problem of data collection in DES studies and advocates a systematic analysis of evidence before using (novel) cognitive tasks intraoperatively [46]. This roadmap provides clear guidelines for future studies to obtain converging and more definite evidence, in which DES results should be combined with the results of lesion-deficit studies (to correlate between tract and function), functional imaging studies (to study the role of a tract in and between functional networks) and structural imaging studies (for accurate visualization and identification of tracts). Importantly, if we want to establish whether or not a certain pathway is indispensable for a particular function, and thus is relevant to neurosurgeons, postoperative (neuropsychological) assessment should be performed to assess the cognitive status of the patient and his or her quality of life in the long run. For future research we should work towards a uniform way of collecting DES findings of subcortical pathways. Only then, we can learn more about the intricate architecture of the brain substantiating executive functions and learn if their underlying pathways are indispensable for normal cognitive functioning.

## Conclusion

The evidence regarding the use of electrical subcortical stimulation mapping for assessment of executive functions and visuospatial awareness is limited. This systematic review provides an indication for several subcortical pathways to be involved in different functions. More importantly, this review reflects the urge for the development of a standardized assessment protocol for intraoperative subcortical mapping of cognitive functions and can function as a stepping stone to reach consensus. This is important for neurosurgical practice to achieve optimal postoperative quality of life and for enhancement of knowledge in neuroscience.

## Supplementary Information

Below is the link to the electronic supplementary material.

Supplementary file1 (DOCX 41 KB)

## Data Availability

Upon request.

## Abbreviations

SLF: Superior longitudinal fasciculus
FAT: Frontal aslant tract
IFG: Inferior frontal gyrus
IFOF: Inferior fronto-occipital fasciculus
FST: Frontostriatal tract
AF: Arcuate fasciculus
SFG: Superior frontal gyrus
SMA: Supplementary motor area
DW-MRI: Diffusion weighted magnetic resonance imaging
DES: Direct electrical stimulation
