# Supplementary material for: A systematic review of the use of subcortical intraoperative electrical stimulation mapping for monitoring of executive deficits and neglect: what is the evidence so far?
Source: Acta Neurochir (Wien). 2021 Oct 21;164(1):177–91. doi: 10.1007/s00701-021-05012-w (PMC8761150; doi:10.1007/s00701-021-05012-w)
Supplement: Supplementary file 1 — Supplementary file1 (DOCX 41 KB) [file 701_2021_5012_MOESM1_ESM.docx]

**Supplemental material**

*Article title:* A systematic review of the use of subcortical intraoperative electrical stimulation mapping for assessment of executive functions: what is the evidence so far?

*Journal name:* Acta Neurochirurgica

*Author names*: Maud J.F. Landers^1,2^, Margriet M. Sitskoorn^2^, Geert-Jan M. Rutten^1^, Emmanuel Mandonnet^3,4,5^, Wouter De Baene^2^

*Affiliations:*

^1.^ Department of Neurosurgery, Elisabeth-TweeSteden Hospital Tilburg, the Netherlands

^2.^ Department of Cognitive Neuropsychology, Tilburg University, Tilburg, the Netherlands

^3^ Université de Paris, Paris, France

^4^ Frontlab, Institut du Cerveau (ICM), CNRS UMR 7225, INSERM U1127, Paris, France

^5^ Service de Neurochirurgie, Hôpital Lariboisière, Paris, France

*Corresponding author*: Maud Janne Francis Landers

*Email address*: m.landers@etz.nl

**Supplemental material 1**

**PUBMED**

(glioma* [tiab] OR brain neoplasms[MeSH Terms] OR brain neoplasm* [tiab] OR glioma[MeSH Terms] OR brain tumor* [tiab]) AND (awake [tiab] OR electric stimul*[tiab] OR electrical stimul*[tiab] OR DES[tiab] OR electric-map* [tiab] OR electrical-map* [tiab] OR intraoperative-map*[tiab] OR brain-map*[tiab] OR brain mapping[MeSH Terms]) AND (subcort*[tiab] OR tract*[tiab] OR fascicul*[tiab] OR fiber*[tiab] OR myelin*[tiab] OR pathway*[tiab] OR white matter[MeSH Terms] OR white matter*[tiab]) AND (neuropsych*[tiab] OR cognit*[tiab] OR Cognition[MeSH Terms] OR Cognitive dysfunction[MeSH Terms] OR Neuropsychological tests[MeSH Terms] OR Executive functions[MeSH Terms] OR neglect* [tiab] OR memory [tiab]))

1-11-2020: 147 results

**EMBASE**(('brain tumor'/exp OR 'glioma'/exp OR ‘brain tumor*’:ti,ab OR glioma*:ti,ab OR ‘brain neoplasm*’:ti,ab) AND ('brain mapping'/exp OR awake:ti,ab OR ‘electric stimul*’:ti,ab OR ‘electrical stimul*’:ti,ab OR DES:ti,ab OR electric-map*:ti,ab OR electrical-map*:ti,ab OR intraoperative-map*:ti,ab OR brain-map*:ti,ab) AND ('white matter'/exp OR subcort*:ti,ab OR tract*:ti,ab OR fascicul*:ti,ab OR fiber*:ti,ab OR myelin*:ti,ab OR pathway*:ti,ab OR ‘white matter*’:ti,ab) AND ('cognition'/exp OR 'cognitive defect'/exp OR 'neuropsychological test'/exp OR executive functions'/exp OR neuropsych*:ti,ab OR cognit*:ti,ab OR neglect*:ti,ab OR memory:ti,ab))

1-11-2020: 293 results

**COCHRANE**(glioma* OR brain neoplasm* OR brain tumor*) AND (awake OR electric stimul* OR electrical stimul* OR DES OR electric-map* OR electrical-map* OR intraoperative-map* OR brain-map*) AND (subcort* OR tract* OR fascicul* OR fiber* OR myelin* OR pathway* OR white matter*) AND (neuropsych* OR cognit* OR executive function* OR neglect OR memory) in Title Abstract Keyword

1-11-2020: 4 results

**WEB OF SCIENCE**TS=(glioma* OR brain neoplasm* OR brain tumor*) AND TS=(awake OR electric stimul* OR electrical stimul* OR DES OR electric-map* OR electrical-map* OR intraoperative-map* OR brain-map*) AND TS=(subcort* OR tract* OR fascicul* OR fiber* OR myelin* OR pathway* OR white matter*) AND TS=(neuropsych* OR cognit* OR executive function* OR neglect OR memory)

1-11-2020: 131 results

**Supplemental material 2**

**Table 2.** The mean 8-item and 12-item MINORS score for the 12 included studies

|  | Burks  [1] | Herbet  [2] | Motomura  [3] | Papagno  [4] | Kinoshita  [5] | Roux  [6] | Thiebaut  [7] | Vallar  [8] | Puglisi  [9] | Mandonnet  [10] | Rolland  [11] |
| --- | --- | --- | --- | --- | --- | --- | --- | --- | --- | --- | --- |
| 1. A clearly stated aim | 2 | 2 | 2 | 2 | 2 | 2 | 2 | 2 | 2 | 2 | 2 |
| 2. Inclusion of consecutive patients | 2 | * | 1 | 1 | * | 1 | * | 1 | 2 | * | 2 |
| 3. Prospective collection of data | 0 | 2 | 0 | 2 | 0 | 2 | 2 | 2 | 2 | 2 | 0 |
| 4. Endpoints appropriate to the aim of the study | 2 | 2 | 1 | 2 | 2 | 2 | 2 | 2 | 2 | 2 | 2 |
| 5. Unbiased assessment of the study endpoint | 0 | 0 | 0 | 0 | 0 | 0 | 0 | 0 | 0 | 0 | 0 |
| 6. Follow-up period appropriate to the aim of the study | 2 | 2 | 2 | 2 | 2 | 2 | 0 | 0 | 2 | 2 | 2 |
| 7. Loss to follow up less than 5% | 2 | * | 2 | 1 | * | 1 | * | 2 | 2 | * | 2 |
| 8. Prospective calculation of the study size | 0 | * | 0 | 0 | * | 0 | * | 0 | 0 | * | 0 |
|  |  |  |  |  |  |  |  |  |  |  |  |
| Total: | 10 | 8 | 8 | 10 | 6 | 10 | 6 | 9 | 12 | 8 | 10 |
|  |  |  |  |  |  |  |  |  |  |  |  |
| *Additional criteria in the case of comparative study* |  |  |  |  |  |  |  |  |  |  |  |
| 9. An adequate control group | 2 |  |  |  | 2 | 2 |  | 2 | 2 |  |  |
| 10. Contemporary groups | 1 |  |  |  | 0 | 2 |  | 2 | 1 |  |  |
| 11. Baseline equivalence of groups | 2 |  |  |  | 1 | 1 |  | 1 | 1 |  |  |
| 12. Adequate statistical analyses | 2 |  |  |  | 2 | 2 |  | 1 | 2 |  |  |
|  |  |  |  |  |  |  |  |  |  |  |  |
| Total: | 17 |  |  |  | 11 | 17 |  | 15 | 18 |  |  |

^1.^ Scores meaning 0 (not reported), 1 (reported but inadequate) or 2 (reported and adequate).
*Case report or case series

**References**

1. Burks, J.D., et al., *A method for safely resecting anterior butterfly gliomas: the surgical anatomy of the default mode network and the relevance of its preservation.* J Neurosurg, 2017. **126**(6): p. 1795-1811.

2. Herbet, G., Y.N. Yordanova, and H. Duffau, *Left Spatial Neglect Evoked by Electrostimulation of the Right Inferior Fronto-occipital Fasciculus.* Brain Topography, 2017. **30**(6): p. 747-756.

3. Motomura, K., et al., *Supratotal Resection of Diffuse Frontal Lower Grade Gliomas with Awake Brain Mapping, Preserving Motor, Language, and Neurocognitive Functions.* World Neurosurgery, 2018. **119**: p. 30-39.

4. Papagno, C., et al., *Mapping the brain network of the phonological loop.* Hum Brain Mapp, 2017. **38**(6): p. 3011-24.

5. Kinoshita, M., et al., *Chronic spatial working memory deficit associated with the superior longitudinal fasciculus: a study using voxel-based lesion-symptom mapping and intraoperative direct stimulation in right prefrontal glioma surgery.* Journal of neurosurgery, 2016. **125**(4): p. 1024-1032.

6. Roux, F.E., et al., *Electrostimulation mapping of spatial neglect.* Neurosurgery, 2011. **69**(6): p. 1218-31.

7. Thiebaut de Schotten, M., et al., *Direct evidence for a parietal-frontal pathway subserving spatial awareness in humans.* Science, 2005. **309**(5744): p. 2226-8.

8. Vallar, G., et al., *Cerebral correlates of visuospatial neglect: a direct cerebral stimulation study.* Hum Brain Mapp, 2014. **35**(4): p. 1334-50.

9. Puglisi, G., et al., *- Frontal pathways in cognitive control: direct evidence from intraoperative stimulation and diffusion tractography.* 2019. **- 142**.

10. Mandonnet, E., et al., *Network-level causal analysis of set-shifting during trail making test part B: A multimodal analysis of a glioma surgery case.* Cortex, 2020. **132**: p. 238-249.

11. Rolland, A., G. Herbet, and H. Duffau, *Awake Surgery for Gliomas within the Right Inferior Parietal Lobule: New Insights into the Functional Connectivity Gained from Stimulation Mapping and Surgical Implications.* World Neurosurgery, 2018. **112**: p. E393-E406.
